# Proportion and related factors of depression and anxiety for inpatients with lung cancer in China: a hospital-based cross-sectional study

**DOI:** 10.1007/s00520-022-06961-3

**Published:** 2022-03-23

**Authors:** Xueqian Wang, Xuejiao Ma, Mo Yang, Yan Wang, Yi Xie, Wei Hou, Ying Zhang

**Affiliations:** Department of Oncology, Guang’anmen Hospital, China Academy of Chinese Medical Sciences, Beijing, 100053 China

**Keywords:** Lung cancer, Depression, Anxiety, Related factors

## Abstract

**Background:**

Lung cancer was often accompanied by depression and anxiety. Nowadays, most investigations for depression and anxiety were concentrated in western medical hospitals, while few related studies have been carried out in the tradition Chinese medicine (TCM) ward. It was necessary to understand the proportion and related factors of depression and anxiety in the inpatients with lung cancer in TCM hospital.

**Methods:**

This study adopted cross-sectional research method, which enrolled a total of 222 inpatients with lung cancer in TCM hospital. PHQ-9 and GAD-7 scales were used to assess depression and anxiety for the inpatients, respectively. Demographic and clinical data were also collected. Statistical methods of the univariate analysis and the multivariate logistic regression model were used.

**Results:**

The proportion of depression and anxiety in the inpatients with lung cancer were 58.1% and 34.2%, respectively. Multivariate logistic regression analysis prompted that the common related factor of depression and anxiety was the symptom of insomnia(odds ratio [OR] 3.274, 95%CI 1.723–6.219; OR 2.201, 95%CI 1.132–4.277). Constipation and gender were the two anther-related factors of depression(OR 1.458, 95%CI 0.372–1.606; OR 1.298, 95%CI 0.151–1.588).

**Conclusion:**

Depression and anxiety were common for the inpatients with lung cancer in TCM hospital. Gender, insomnia, and constipation were related factors for depression, and insomnia was related factor for anxiety. Therefore, medical workers should pay close attention to the emotional changes of these high-risk patients and intervene the symptoms as early as possible.

## Introduction

Lung cancer was the most common cancer and the leading cause of cancer death in China and worldwide [1]. In recent years, the incidence of lung cancer had increased substantially in China and was likely to continue to rise in the next few decades [2]. Most patients with lung cancer usually confronted a limited life span and had to receive surgery, chemical radiotherapy, targeted therapy, and other treatments. Benefiting from the development of treatment methods, more and more patients with lung cancer could receive more treatments to survive longer. However, these treatments and the disease of lung cancer itself often caused many uncomfortable symptoms, impaired physical function, financial burden, social tension, and psychological disorders. Besides the efficacy of cancer treatment, these issues should also arouse our widespread concern. In particular, the low cure rate, limited overall survival, and continuous therapies leaded the patients to experience considerable psychological disorder, which should arouse widespread attention from doctors and nurses engaged in clinical work of lung cancer [3].

Nowadays, the psychological disorder of patients with cancer had been determined by National Comprehensive Cancer Network (NCCN) guidelines as the sixth vital characteristic besides body temperature, pulse, breathing, blood pressure, and pain [4]. Depression and anxiety as the most common psychological disorders also most troubled the patients with lung cancer [5]. Extensive studies had shown that the incidence of depressive and anxious in the patients with lung cancer were 38.9–57.1% and 20.9–43.5%, respectively [6–8]. In addition, the research revealed that cancer patients with depression and anxiety had not only a worse quality of life but also longer hospital stay and higher costs [9, 10]. And psychological disorder of depression and anxiety had been regarded as a predictor of shorter survival in patients with lung cancer [11]. Therefore, it was very meaningful to investigate the symptoms of depression and anxiety in patients with lung cancer and explored the influencing factors of them in order to better understand and respond.

Nowadays, we had obtained the data related to depression and anxiety in patients with lung cancer, which were mainly from western medical hospitals [6, 7]. Only a few investigations on depression and anxiety on patients with lung cancer were carried out in TCM hospitals but only in outpatients [8]. It was widely known that Chinese medicine therapy was different from the western medicine, and the characteristics of outpatients and inpatients were also very different. This study intended to use internationally recognized scales to assess the proportion of depression and anxiety in the inpatients with lung cancer of TCM hospital and to determine the related risk factors of depression and anxiety.

## Methods

### Participants

The cross-sectional study recruited patients with lung cancer who were hospitalized in the Oncology Department of Guang’anmen Hospital, a Grade 3A TCM hospital in Beijing, between January 1 and December 31, 2019. The diagnosis and tumor node metastasis (TNM) staging of lung cancer referred to “Chinese guidelines for diagnosis and treatment of primary lung cancer 2018 (English version)” [12].

Inclusion criteria were (i) diagnosed with primary bronchial lung cancer by pathology and/or cytology, (ii) aware of having lung cancer, (iii) hospitalized in the oncology department of Guangʼanmen Hospital for the first time in 2019, (iv) aged ≥18 years, (v) can communicate with clinicians and cooperate with investigation, and (vi) can understand the questions included in the questionnaire.

Exclusion criteria were (i) uncertain cancer diagnosis, (ii) schizophrenia or other psychiatric disorders, (iii) acute or unstable complications, (iv) poor compliance and unwilling to complete data filling, and (v)cognitive impairment.

### Procedures

The proposal was approved by the Ethics Committee of Guang’anmen Hospital, China Academy of Chinese Medical Sciences (reference number:2016-048-KY-02). Potential participants were approached and invited to this study on the first day when they were admitted to the hospital ward. This was a convenience sample. The study was conducted in the oncology ward of Guangʼanmen Hospital. After the inclusion criteria of patients was determined, the method and purpose of the research were explained to them. After the patients provided written informed consent, their information was collected. The study was conducted in compliance with the Declaration of Helsinki.

### Measurements

All evaluation data would be collected on the first day of patientsʼ hospitalization:

1. General information including name, age, gender, and medical insurance.
2. Clinical information including disease course(days), treatment method (surgery, chemotherapy, radiotherapy, targeted therapy, immunotherapy), BMI, NRS score, KPS score, pathological classification, TNM staging, tobacco smoking, and other chronic comorbid conditions information.
3. Clinical symptoms including poor appetite, cough, constipation, diarrhea, and insomnia.
4. Observation indexes were PHQ-9(9-Item Patient Health Questionnaire) [13] and GAD-7(7-Item Generalized Anxiety Disorder ) [14] scales score, which had been validated to Chinese for evaluating the depression and anxiety, respectively [15].

General information and partial clinical information were gathered from the medical records available. Another partial clinical information, clinical symptoms and observation indexes of two scales score were obtained from the patients directly.

### Depression

The PHQ-9 was a 9-item scoring scale designed and validated for diagnosis and grading depression based on DSM-IV criteria, including the following aspects: (1) anhedonia; (2) depressed mood; (3) trouble sleeping; (4) feeling tired; (5) change in appetite; (6) guilt, self-blame, or worthlessness; (7) trouble concentrating; (8) feeling slowed down or restless; and (9) thoughts of being better off dead or hurting oneself [16]. Symptoms are rated using a 4-point scale (0, never; 1, several days; 2, more than half the time; 3, nearly every day) regarding the past 2 weeks experienced. The overall scores ranged from 0 to 27. Total score 0–4 points indicated the lack of any depression disorder, 5–9 indicated mild depression,10–14 indicated moderate depression, 15–19 indicated moderate and severe depression, and 20–27 indicated severe depression.

### Anxiety

GAD-7 [17] was a questionnaire designed to assess anxiety symptoms. Patients were invited to answer 7 questions assessing past two-weeks period.

Questions:

1. Feeling nervous, anxious, or on edge.
2. Not being able to stop or control worrying.
3. Worrying too much about different things.
4. Trouble relaxing.
5. Being so restless that was hard to sit still.
6. Becoming easily annoyed or irritable.
7. Feeling afraid as if something awful might happen.

Four alternatives are offered: (A) Not at all; (B) Several days; (C) More than half the days; and (D) Nearly every day. Scores could range from 0 to 21. Total score 0–4 points indicated no anxiety, 5–9 indicated mild anxiety, 10–13 indicated moderate anxiety, 14–18 indicated moderate and severe anxiety, and 19–21 indicated severe anxiety.

### Chronic comorbid conditions and physical symptom burden

To analyze the chronic comorbid conditions and physical symptoms associated with depression and anxiety, four common chronic medical conditions were added: hypertension, diabetes mellitus, coronary heart disease, and hyperlipidemia. And five common physical symptoms were added: insomnia, cough, constipation, diarrhea, and poor appetite, which are assessed by Guidelines for clinical research of Traditional Chinese Drug Research [18]^.^

### Statistical analyses

The SPSS 24.0 software was used for statistical analysis of all data, using a two-sided difference test. *P* ≤ 0.05 is considered statistically significant. Descriptive statistics for both continuous (frequencies, mean, standard deviation) and categorical variables (frequencies, percentages) were calculated. Comparisons between depression/anxiety and non-depression/non-anxiety groups were performed in a one-way analysis of variance. To identify significant factors associated with depression and anxiety inpatients with lung cancer, a multivariate logistic regression model was used after univariate analysis.

## Results

### Characteristics of all participants

**Fig. 1:**
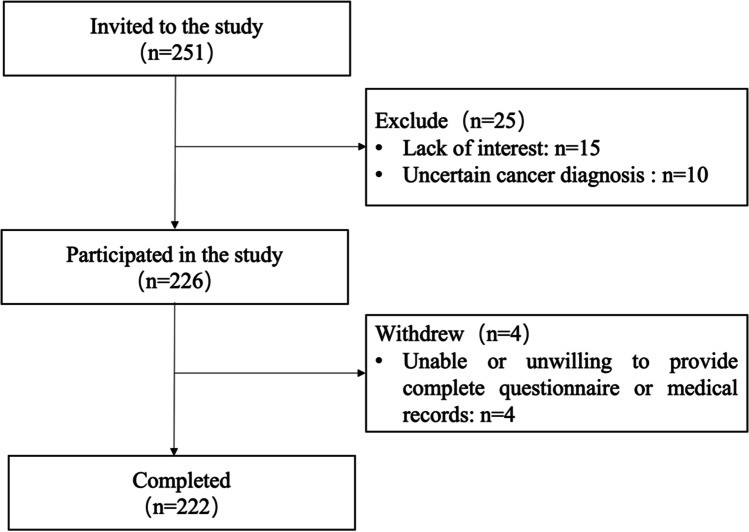
Flowchart of the study recruitment

A total of 251 inpatients with lung cancer met the inclusion criteria. After excluding 15 participants not interested in the study, 10 patients with uncertain cancer diagnosis, and 4 patients who did not complete the questionnaire, 222 patients completed the study (Fig. 1). According to the rough estimation method of sample size, the sample size was 5–10 times the number of variables [19, 20]. The number of cases enrolled in this study throughout 2019 should meet the requirements. The general information, clinical information, and other symptoms data collected from 222 inpatients were presented in Table 1.

**Table 1:** Characteristics of all participants

Variable	Frequency (***N***=222)	Percentage (%)	Mean (SD)	Range
Gender				
Male	138	62.2		
Female	84	37.8		
Age(years)			66.36(9.979)	37–89
Medical insurance				
Without medical insurance	21	9.5		
With medical insurance	201	90.5		
TNM staging				
Stage I	13	5.9		
Stage II	19	8.6		
Stage III	46	20.7		
Stage IV	144	64.9		
Pathological type				
Adenocarcinoma	86	38.7		
Squamous cell carcinomas	54	24.3		
Small cell lung cancer	37	16.7		
Neuroendocrine carcinoma	4	1.8		
Sarcomatoid carcinoma	4	1.8		
Others	37	16.7		
NRS(scores)			2(2.378)	0–10
KPS(scores)			81.53(12.777)	30–100
BMI			23.14(2.526)	13.67–30.05
Disease course(days)			399.76(661.089)	2–4020
Surgery				
Yes	57	25.7		
No	165	74.3		
Chemotherapy				
Yes	83	37.4		
No	139	72.6		
Radiotherapy				
Yes	25	11.3		
No	197	88.7		
Targeted therapy				
Yes	40	18		
No	182	82		
Immunotherapy				
Yes	6	2.7		
No	216	97.7		
Hypertension				
Yes	98	44.1		
No	124	55.9		
Diabetes mellitus				
Yes	48	21.6		
No	176	78.4		
Coronary heart disease				
Yes	46	20.7		
No	176	79.3		
Hyperlipidemia				
Yes	30	13.5		
No	192	86.5		
Tobacco smoking				
Yes	114	51.4		
No	108	48.6		
Poor appetite				
Yes	143	64.4		
No	79	35.6		
Cough				
Yes	188	84.7		
No	34	15.3		
Constipation				
Yes	54	24.3		
No	168	75.7		
Diarrhea				
Yes	6	2.7		
No	216	97.3		
Insomnia				
Yes	103	53.6		
No	119	46.4		

### Proportion of depression and anxiety of inpatients

Among 222 inpatients with lung cancer, 129 were determined to be depressed including 72 with mild depression, 37 with moderate depression, 16 with moderate and severe depression, and 4 severe depressions. And 76 was determined to be anxious including 57 with mild anxiety, 12 with moderate anxiety, 6 with moderate and severe anxiety, and 1 with severe anxiety.

### Univariate analysis of factors in depression and anxiety

Results of univariate analysis on depression showed that there were significant differences in variables of gender, KPS scores, NRS scores, insomnia, poor appetite, surgery, and tobacco smoking. For anxiety, there were significant differences in variables of gender, NRS scores, BMI, disease course, insomnia, constipation, and tobacco smoking (Tables 2 and 3).

**Table 2:** Univariate analysis of factors in depression

Variable	Depression ***N***=129	Non-depression ***N***=93	X^2^	P
Gender			6.643	0.010*
Male	71	67		
Female	58	26		
Age (years)			1.588	0.452
≤45	3	5		
46–64	49	32		
≥65	77	56		
Medical insurance			1.691	0.142
Without medical insurance	11	10		
With medical insurance	118	83		
TNM staging			1.873	0.599
Stage I	7	6		
Stage II	11	8		
Stage III	23	23		
Stage IV	88	56		
Pathological type			9.657	0.29
Adenocarcinoma	54	32		
Squamous cell carcinomas	30	24		
Small cell lung cancer	23	14		
Neuroendocrine carcinoma	2	2		
Sarcomatoid carcinoma	4	0		
Others	16	21		
NRS(scores)	2.33	1.56	5.691	0.018*
KPS(scores)	79.34	84.57	9.394	0.002*
BMI	22.92	23.46	1.276	0.26
Disease course(days)	444.56	337.61	1.417	0.235
Surgery			4.588	0.032*
Yes	40	17		
No	89	76		
Chemotherapy			0.248	0.619
Yes	50	33		
No	79	60		
Radiotherapy			0.432	0.511
Yes	13	12		
No	116	81		
Targeted therapy			0.387	0.534
Yes	25	15		
No	104	78		
Immunotherapy			1.612	0.204
Yes	5	1		
No	124	92		
Hypertension			1.917	0.166
Yes	62	36		
No	67	57		
Diabetes mellitus			0.486	0.485
Yes	30	18		
No	99	75		
Coronary heart disease			1.228	0.67
Yes	28	18		
No	101	75		
Hyperlipidemia			0.49	0.921
Yes	18	12		
No	111	81		
Tobacco smoking			3.886	0.049*
Yes	59	55		
No	70	38		
Poor appetite			13.842	<0.001**
Yes	70	73		
No	59	20		
Cough			0.082	0.775
Yes	110	78		
No	19	15		
Constipation			3.177	0.075
Yes	37	17		
No	92	76		
Diarrhea			1.612	0.204
Yes	5	1		
No	124	92		
Insomnia			21.881	<0.001**
Yes	77	26		
No	52	67		

*P<0.05,**P<0.01

**Table 3:** Univariate analysis of factors in anxiety

Variable	Anxiety ***N***=76	Non-anxiety ***N***=146	X^2^	P
Gender			7.267	0.007^**^
Male	38	100		
Female	38	46		
Age (years)			4.626	0.099
≤45	2	6		
46–64	35	46		
≥65	39	94		
Medical insurance			0.766	0.259
Without medical insurance	9	12		
With medical insurance	67	134		
TNM staging			1.175	0.759
Stage I	6	7		
Stage II	7	12		
Stage III	14	32		
Stage IV	49	95		
Pathological type			6.870	0.551
Adenocarcinoma	37	49		
Squamous cell carcinomas	14	40		
Small cell lung cancer	11	26		
Neuroendocrine carcinoma	1	3		
Sarcomatoid carcinoma	2	2		
Others	12	25		
NRS(scores)	2.76	1.61	66.512	0.001^**^
KPS(scores)	79.34	82.67	3.431	0.065
BMI	22.45	23.50	4.516	0.035^*^
Disease course(days)	530.95	331.47	4.626	0.033^*^
Surgery			2.111	0.146
Yes	24	33		
No	52	113		
Chemotherapy			0.029	0.885
Yes	29	54		
No	47	92		
Radiotherapy			0.062	0.803
Yes	8	17		
No	68	129		
Targeted therapy			2.512	0.113
Yes	18	22		
No	58	124		
Immunotherapy			2.881	0.09
Yes	4	2		
No	72	144		
Hypertension			0.527	0.468
Yes	31	67		
No	45	79		
Diabetes mellitus			0.242	0.623
Yes	15	33		
No	61	113		
Coronary heart disease			0.372	0.542
Yes	14	32		
No	62	114		
Hyperlipidemia			0.882	0.348
Yes	8	22		
No	68	124		
Tobacco smoking			3.955	0.047^*^
Yes	32	82		
No	44	64		
Poor appetite			3.095	0.079
Yes	33	46		
No	43	100		
Cough			0.415	0.52
Yes	66	122		
No	10	24		
Constipation			7.878	0.005^**^
Yes	27	27		
No	49	119		
Diarrhea			0.681	0.409
Yes	3	3		
No	73	143		
Insomnia			11.085	0.001^**^
Yes	47	56		
No	29	90		

*P<0.05,**P<0.01

### Multivariate analysis of factors in depression and anxiety

In univariate analysis, we selected variables with *P*<0.1 for binary regression analysis. For depression, the variables were gender, KPS scores, NRS scores, insomnia, poor appetite, surgery, tobacco smoking, and constipation. For anxiety, the variables were gender, NRS scores, BMI, disease course, insomnia, constipation, tobacco smoking, age, KPS scores, immunotherapy, and poor appetite.

**Fig. 2:**
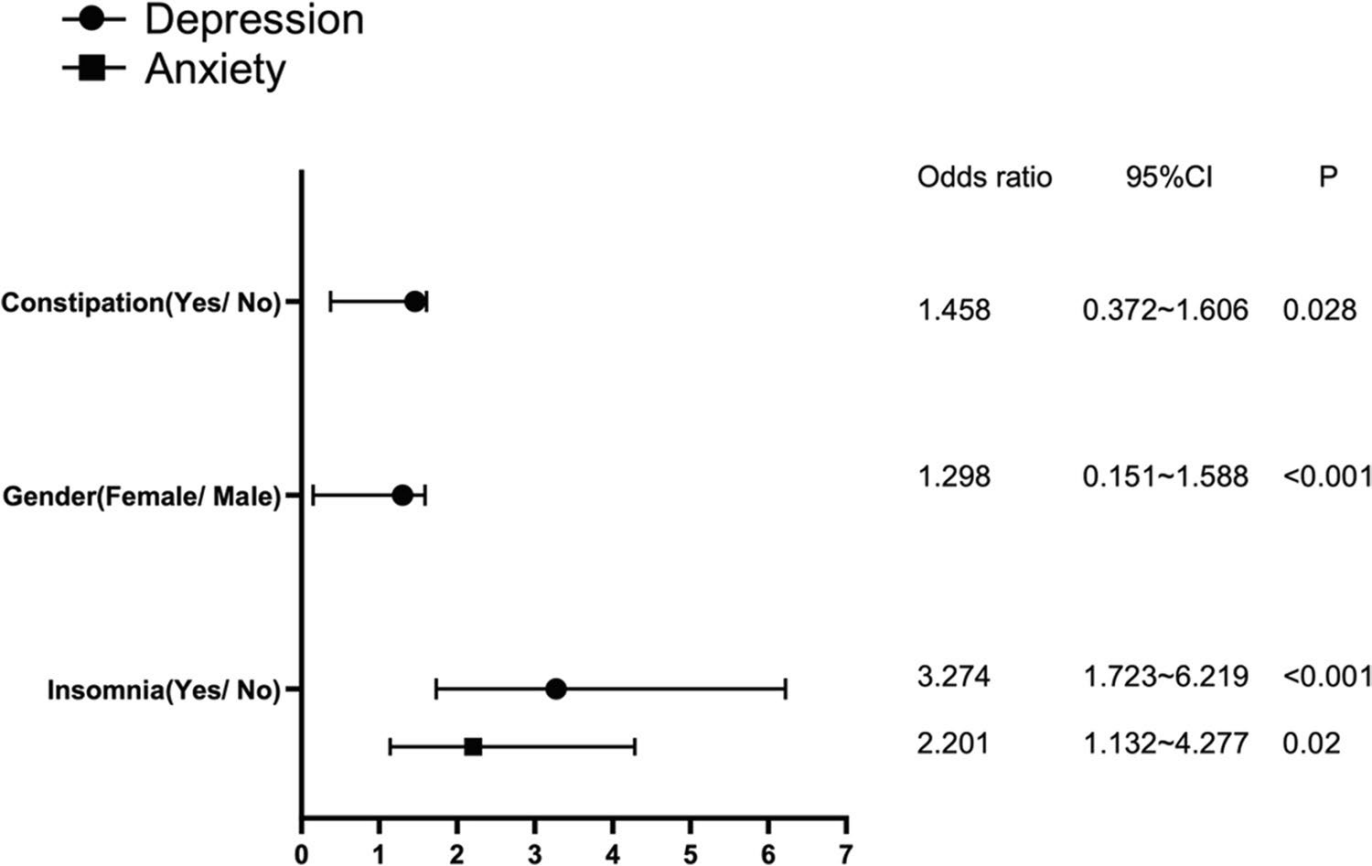
Multivariate analysis of factors in depression and anxiety

The significant related factors of depression and anxiety were shown in Fig. 2. Patients with insomnia were 3.274 times more likely to suffer from depression than those without insomnia and 2.201 times more likely to suffer from anxiety than those without insomnia. Female patients were 1.298 times more likely to be depressed than male. Patients with constipation were 1.458 times more likely to be depressive than non-constipated patients.

## Discussion

The study was aimed to explore the proportion of depression and anxiety in the inpatients with lung cancer in TCM hospital and further to explore the related factors of depression and anxiety. The patients with lung cancer hospitalized for the first time in the oncology department of Guangʼanmen hospital throughout 2019 were recruited. And the characteristics of the inpatients related to depression and anxiety were investigated. It was found that the proportion of depression accounted for 58.1% and moderate or even more serious depression accounted for 25.7%. The proportion of anxiety accounted for 34.2% and moderate or even more serious anxiety accounted for 8.6%.

For depression, regarding the prevalence of depression in the patients with lung cancer in China, our finding was higher than the researches of Xinmiao Wei (53.71%,in Guangxi Province) [21], Guixiang Liu (43.3%,in Jiangsu Province) [22],and Hao Sun (48%,in Shanghai City) [23] et al. in western medical hospitals and even than slightly higher than the X Yan' research(57.1%) [8] on outpatients in Guangʼanmen hospital. This might be related to the characteristics of patients who seek hospitalization treatment in TCM hospital. In China, most cancer patients were usually diagnosed in western medical hospitals and treated with all kinds of modern medical therapies. TCM was not usually considered until patients could not tolerate modern medical treatment or modern medical treatment failed to work. Patients in TCM hospitals usually had a longer course of disease and worse condition than those in western hospitals, which would lead to more depression in TCM hospital. This was basically consistent with Seul Ki Parkʼresearch results [24], which suggested that patients would have a clearer understanding to their condition with the extension of the course of disease. When the disease progressed but not be significantly controlled or adverse reactions due to treatment appeared, the patients' depression would increase. Moreover, inpatients often came from outpatients whose diseases needed further treatment. Considering that data was collected on the first day of patientsʼ hospitalization, in this study the proportion of depression in the inpatients was only 1% higher than that in the outpatients [8].

The proportion of anxiety in the patients with lung cancer from western medical hospitals was 50.71–89.4% [25–27].Comparing with the previous investigations, the proportion of anxiety was reported in our study was lower. This might be related to the fact that patients treated in TCM hospitals generally were usually diagnosed or treated in western medical hospitals in the past. Research showed that anxiety was the main psychological disorder in the early stage of lung cancer. As time went on, anxiety would gradually decrease, which reflected the adaptation process to life-threatening [28]. In addition, some studies also suggested that patientsʼ anxiety was obvious in the early and middle stages of cancer treatment, but the anxiety in the later stage generally could not increase or even decrease [29]. The proportion of anxiety of the inpatients with lung cancer was lower than outpatients [8] in TCM hospital (34.2% vs 43.5%), which might be related to the fact that the patients who had been determined to be able to hospitalize could obtain certain psychological comfort and reduce anxiety [30].

In the investigation, 53.6% of inpatients suffered from insomnia. The study result showed that both anxiety and depression were obviously associated with the symptoms of insomnia. Previous studies had also shown that patients with long-term insomnia would feel helpless and irritable and were more prone to feel depressed and anxious [31]. And longitudinal studies had suggested that cancer patients with persistent insomnia had a higher risk of depression and anxiety [32–36]. These results indicated that insomnia was a key-related risk factor for the depression and anxiety.

In addition, this study also found that both gender and constipation were the related factors for depression. In the study, the gender of female was more likely to have depression, which was 1.458 times that of male. This result had also been explained in many other studies [37]. However, there were few reports on the relationship between depression and constipation in patients with lung cancer. In clinic, inpatients often suffered from constipation. It might be related to the decrease of activity after hospitalization or related to the use of opioid painkillers because of cancer pain [38]. This suggested that we should pay more attention to the psychological status of patients with constipation. In clinic, once the inpatients had insomnia or constipation, doctors should actively improve them and strengthen psychological counseling.

The advantage of this study was to specifically investigate the proportion of depression and anxiety in the patients with lung cancer hospitalized in TCM hospital and explore the related risk factors.

## Study limitations

Due to the cross-sectional design of the analysis, this study could not be determined that the depression or anxiety and related symptoms appeared before or after diagnosis of lung cancer and changed throughout the course of the disease. Consequently, related longitudinal studies were necessary to carry out further. In addition, due to insufficient funds resulting in the limitation of sample size, the correlation analysis was not further carried out between the severity of depression/anxiety and the severity of symptoms. In the future, the sample size should be expanded for further analysis. Thirdly, due to limited research funds and time, only five common clinical symptoms of inpatients were selected in this study, which was a deficiency. Finally, this study was only a single-center study, which might lead to certain selection bias.

## Clinical implications

This study showed that the inpatients with lung cancer in TCM hospital had a high proportion of depression and anxiety. Meanwhile, the possible reasons for the difference of the proportion of depression and anxiety among the inpatients in TCM hospital, the patients in western medical hospital and the outpatients in TCM hospital were analyzed. This study also showed gender, insomnia, and constipation were related factors for depression, and insomnia was related factor for anxiety. Conclusions indicated that the psychological disorders of inpatients with lung cancer in TCM hospital should be paid more attention to. And the common clinical symptoms such as insomnia and constipation should also be concerned as early as possible.

## Conclusion

This study demonstrated that depression and anxiety were very common in the patients with lung cancer hospitalized in TCM hospital. The variables of gender, insomnia, and constipation were the independently related factors for the depression, and insomnia was for anxiety. Therefore, medical workers should pay close attention to the emotional changes of these high-risk patients.

## Data Availability

Not applicable.

## References

[1] Bray F, Ferlay J, Soerjomataram I, Siegel RL, Torre LA, Jemal A (2018). Global cancer statistics 2018: GLOBOCAN estimates of incidence and mortality worldwide for 36 cancers in 185 countries. CA: a cancer journal for clinicians.

[2] Chen W, Zheng R, Baade PD, Zhang S, Zeng H, Bray F, Jemal A, Yu XQ, He J (2016). Cancer statistics in China, 2015. CA: a cancer journal for clinicians.

[3] van de Wiel M, Derijcke S, Galdermans D, Daenen M, Surmont V, De Droogh E, Lefebure A, Saenen E, Vandenbroucke E, Morel AM, Sadowska A, van Meerbeeck JP, Janssens A (2021). Coping strategy influences quality of life in patients with advanced lung cancer by mediating mood. Clin Lung Cancer.

[4] Holland JC, Bultz BD, National comprehensive Cancer Network (NCCN), (2007). The NCCN guideline for distress management: a case for making distress the sixth vital sign. Journal of the National Comprehensive Cancer Network.

[5] Rujun Z, Junying Li (2011). Research progress in the evaluation and treatment of psychological distress for cancer patients. West China Medicine.

[6] Jung JY, Lee JM, Kim MS, Shim YM, Zo JI, Yun YH (2018). Comparison of fatigue, depression, and anxiety as factors affecting posttreatment health-related quality of life in lung cancer survivors. Psychooncology.

[7] Polański J, Chabowski M, Chudiak A, Uchmanowicz B, Janczak D, Rosińczuk J, Mazur G (2018). Intensity of Anxiety and Depression in Patients with Lung Cancer in Relation to Quality of Life. Adv Exp Med Biol.

[8] Yan X, Chen X, Li M, Zhang P (2019). Prevalence and risk factors of anxiety and depression in Chinese patients with lung cancer:a cross-sectional study. Cancer management and research.

[9] Gu W, Xu YM, Zhong BL (2018). Health-related quality of life in Chinese inpatients with lung cancer treatedin large general hospitals: across-sectional study. BMJ Open.

[10] Gu W, Xu YM, Zhu JH, Zhong BL (2017). Depression and its impact on health-related quality of life among Chinese inpatients with lung cancer. Oncotarget.

[11] Pirl WF, Greer JA, Traeger L, Jackson V, Lennes IT, Gallagher ER, Perez-Cruz P, Heist RS, Temel JS (2012). Depression and survival in metastatic non-small-cell lung cancer: effects of early palliative care. Journal of Clinical Oncology.

[12] Health Commission of PRC National (2019). Chinese guidelines for diagnosis and treatment of primary lung cancer 2018 (English version). Chin J Cancer Res.

[13] Hartung TJ, Friedrich M, Johansen C, Wittchen HU, Faller H, Koch U, Brähler E, Härter M, Keller M, Schulz H, Wegscheider K, Weis J, Mehnert A (2017). The Hospital Anxiety and Depression Scale (HADS) and the 9-item Patient Health Questionnaire (PHQ-9) as screening instruments for depression in patients with cancer. Cancer.

[14] Peres M, Mercante J, Tobo PR, Kamei H, Bigal ME (2017). Anxiety and depression symptoms and migraine: a symptom-based approach research. J Headache Pain.

[15] Wang Beidi (2013) Application research of PHQ-9 and GAD-7 in patients with malignant tumors (Master's thesis, Central South University). (in China)

[16] Kroenke K, Spitzer RL, Williams JB (2001). The PHQ-9: validity of a brief depression severity measure. J Gen Intern Med.

[17] Spitzer RL, Kroenke K, Williams JB, Löwe B (2006). A brief measure for assessing generalized anxiety disorder: the GAD-7. Arch Intern Med.

[18] Zheng XY (2002). Guidelines for clinical research of Traditional Chinese Drug Research[M].

[19] Osman A, Barrios FX, Gutierrez PM, Kopper BA, Merrifield T, Grittmann L (2000). The Pain Catastrophizing Scale: further psychometric evaluation with adult samples. J Behav Med.

[20] Zhu GH, Li J, Li J, Xu BW, Wang HP, Wang XM, Hu JQ, Dai MH (2021). The characteristics and related factors of insomnia among postoperative patients with gastric cancer: a cross-sectional survey. Support Care Cancer.

[21] Xinmiao W (2013). Logistic regression analysis of depression and related risk factors in patients with lung cancer. Medical Review.

[22] Guixiang L, Wenjuan J, Xiaoping S (2020). Risk factors of depression in hospitalized patients with lung cancer and their impact on quality of life. Journal of Clinical Pulmonology.

[23] Sun Hao Lu, Xiaofang WW, Yi L, Yuyuan Z, Wenfeng Z, Tong Su (2018). The correlation and influencing factors between quality of life and anxiety and depression of lung cancer inpatients in a hospital in Shanghai. Med Soc.

[24] Park SK, Min YH, Lee SB (2021). Longitudinal trends in illness perception and depression during adjuvant breast cancer endocrine therapy: a prospective observational study. Healthcare (Basel, Switzerland).

[25] Zang Yu, Junmin Z, Weijing Qi (2018). The symptom group of lung cancer patients and its correlation with anxiety and depression. Nurs Res.

[26] Longfang P, Xiangmei Y, Yueling H (2012). Research on related factors of anxiety in patients with lung cancer. Nurs Res.

[27] Yumin Z, Mining L, Deng Lu (2009). Investigation and analysis of anxiety and depression in hospitalized patients with lung cancer. Contemporary Nurses (Academic Edition).

[28] Heszen-Niejodek I, Gottschalk LA, Januszek M (1999). Anxiety and hope during the course of three different medical illnesses: a longitudinal study. Psychother Psychosom.

[29] Deshields T, Tibbs T, Fan MY, Bayer L, Taylor M, Fisher E (2005). Ending treatment: the course of emotional adjustment and quality of life among breast cancer survivors immediately following radiation therapy. Supportive Care in Cancer.

[30] Niedzwiedz CL, Knifton L, Robb KA, Katikireddi SV, Smith DJ (2019). Depression and anxiety among people living with and beyond cancer: a growing clinical and research priority. BMC Cancer.

[31] Jarrin DC, Chen IY, Ivers H, Morin CM (2014). The role of vulnerability in stress-related insomnia, social support and coping styles on incidence and persistence of insomnia. J Sleep Res.

[32] Ford DE, Kamerow DB (1989). Epidemiologic study of sleep disturbances and psychiatric disorders. An opportunity for prevention?. JAMA.

[33] Breslau N, Roth T, Rosenthal L, Andreski P (1996). Sleep disturbance and psychiatric disorders: a longitudinal epidemiological study of young adults. Biol Psychiat.

[34] Chang PP, Ford DE, Mead LA, Cooper-Patrick L, Klag MJ (1997). Insomnia in young men and subsequent depression. The Johns Hopkins Precursors Study. American Journal of Epidemiology.

[35] Gillin JC (1998). Are sleep disturbances risk factors for anxiety, depressive and addictive disorders?. Acta Psychiatr Scand Suppl.

[36] Livingston G, Blizard B, Mann A (1993). Does sleep disturbance predict depression in elderly people? A study in inner London. The British Journal of General Practice.

[37] Linden W, Vodermaier A, Mackenzie R, Greig D (2012). Anxiety and depression after cancer diagnosis: prevalence rates by cancer type, gender, and age. J Affect Disord.

[38] Chokhavatia S, John ES, Bridgeman MB, Dixit D (2016). Constipation in Elderly Patients with Noncancer Pain: Focus on Opioid-Induced Constipation. Drugs Aging.

